# Evidence for Protective Effects of Peer Play in the Early Years: Better Peer Play Ability at Age 3 Years Predicts Lower Risks of Externalising and Internalising Problems at Age 7 Years in a Longitudinal Cohort Analysis

**DOI:** 10.1007/s10578-022-01368-x

**Published:** 2022-06-14

**Authors:** Yiran Vicky Zhao, Jenny Louise Gibson

**Affiliations:** Play and Communication Lab, Centre for Research on Play in Education, Development and Learning, Faculty of Education, University of Cambridge, 184 Hills Road, Cambridge, CB2 8PQ UK

**Keywords:** Play, Peer relationships, Mental health, Temperament, Protective factors

## Abstract

Peer play ability may be a protective factor against childhood mental health difficulties but there is lack of empirical evidence to support this hypothesis. We conducted longitudinal structural equation modelling study over a population cohort (N = 1676) to examine the effect of age 3 peer play ability on children’s age 7 mental health outcomes (measured by the Strengths and Difficulties Questionnaire subscales). We modelled effects for the entire population and two sub-groups at high-risk for mental health problems based on age 3 temperament. Controlling for demographic variables, temperament, maternal distress, play with parents and number of siblings, better peer play ability at age 3 years predicted lower risk of problems on all 4 SDQ subscales at age 7 years for the general population. For the low-persistence subgroup, better peer play ability at age 3 predicted lower risk of age 7 hyperactivity, emotional and peer problems, whereas better peer play ability at age 3 predicted only lower risk of age 7 hyperactivity for the high-reactivity group. Taken together our results provide evidence that supports the hypothesis that early peer play ability may be a protective factor against later mental health difficulties. We conclude that further research aimed at establishing causation is worth pursuing.

**Supplementary Information:**

The online version contains supplementary material available at 10.1007/s10578-022-01368-x.

## Introduction

Social relationships have a profound influence upon mental health. Individuals experiencing more positive reciprocal relationships are not only likely to experience good mental health themselves, but also to contribute to the mental health of people in their social networks [1]. For children and young people, relationships with parents and caregivers are undoubtedly important predictors of mental health [2], however the influence of peers increases as children mature [3]. In many cultures, preschool is often the first time that young children encounter large groups of same aged peers. The early years may therefore be a sensitive period for building the foundations of social competence that set the stage for later social development such as building strong, reciprocal friendships.

Play is a universally observed phenomenon in the early years of a child’s development and yet its potential developmental functions in social development and emotional regulation are often overlooked when considering mental health [4]. There is a developing evidence base on this topic, but it is at an early stage. For example, Dodd and Lester [5] have linked adventurous play to anxiety management, and Gray [6] has theorised a causal relation between play deprivation and increased psychopathology. Peer play is often considered an essential context for improving children’s emerging social skills and the earliest opportunities to engage in it tend to correspond with the sensitive period for building foundational friendship skills [7].

Despite friendships being central to children’s social lives in early childhood, Schwartz and Badaly [8] argue that research on children’s psychological and behavioural problems tends to focus on the influence of peer rejection, victimisation and friendlessness. Furthermore, while friendship has received some attention in the literature, there is a dearth of empirical studies examining the role of peer play in mental health. However, there are some preliminary findings in this area, Toseeb and colleagues [9] showed that while controlling for factors such as children’s friendships and prosociality between age 7 and 9, children’s social play competences at age 7 significantly predicted lower levels of internalising and externalising problems at age 11 in a UK general population sample. Similarly, Shim and Lim [10] found that negative peer play behaviours reported by preschool teachers at age 4 predicted higher levels of internalising and externalising problems at age 6 in a Korean population after controlling for children’s temperament. Thus, it is likely that peer play abilities can be added to the biopsychosocial models that predict risks for internalising and/or externalising problems, which so far have focused upon early risks associated with heritability, sociodemographic background, parent–child relationships and the interactions between these factors [2]. Given emphasis on early, preventative intervention for mental health outcomes, we investigate predictors in the preschool period. We explore outcomes for mental health in middle childhood, as at this age children are significantly affected by peer relationships and mental health at this age can have a long-lasting impact on psychosocial adjustment [11, 12].

## Conceptualising Peer Play Ability

Those with the strongest views on the evolutionary functions of social play behaviour argue for play as a distinct construct, separable from other socially oriented instincts and behaviour [13, 14]. At the other end of the spectrum, peer play ability is conceptually subsumed within social behaviour more generally and not treated as a distinct phenomenon of interest, in the same way that, for example, bullying and victimisation have been treated in the mental health literature [15].

In the present study, we have taken a position somewhere between these poles. We conceptualise peer play ability as a latent construct reflecting the overall skill and propensity for children to engage with peers in activities that are intrinsically motivating and carried out for inherent enjoyment. Peer play may involve children agreeing the goals of the activity and may involve suspension of reality, for example by setting up shared pretence or rule-governed games with peers. While this approach does not rule out peer play ability as a consequence of a more general social competence, we do suggest that there is value in conceptualising play with peers as a unique, developmentally beneficial way to put one’s social abilities to work. Thus, we are interested in quality of peer play experience not simply quantity (e.g. frequency, duration or variety). We focus on the dynamic nature and playful intent of peer play behaviour, rather than specific interactional forms and so it is different from Parten’s [16] categorisation of peer play into the sequential development of parallel, associative and cooperative play, which has been challenged on the grounds that it does not reflect the variety and complexity of peer play development (see Howes and Matheson [17]).

**Fig. 1:**
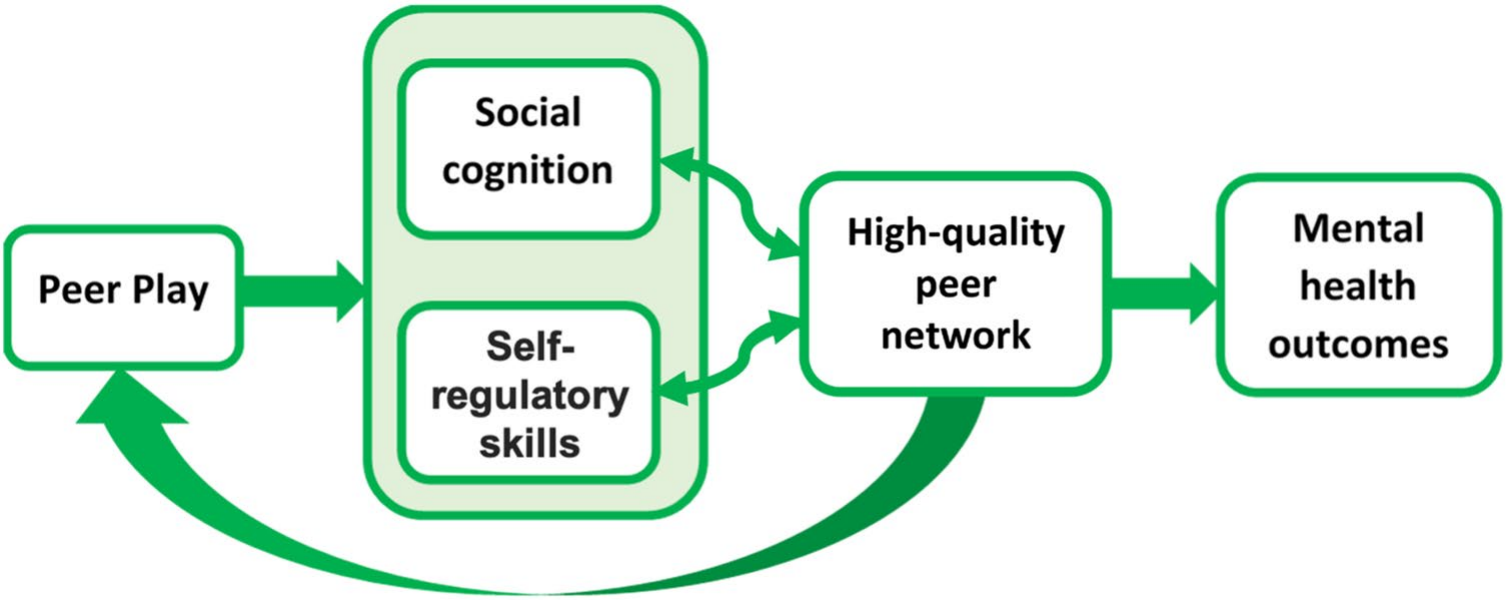
Schematic diagram showing how peer play may influence mental health. Successful peer play builds underlying cognitive skills in social and self-regulatory domains, which in turn help children to form high-quality peer relational networks (including stable relationships and reciprocal friendships) that support mental health. High-quality peer networks may in turn engender more peer play opportunities, creating a virtuous cycle

In addition, our conceptualisation of peer play competence is also different from the widely used Penn Interactive Peer Play Scale that explores children’s social behaviours within peer play including play disruption and play disconnections [18]. Since children’s engagement in play activities is determined by their own preferences [19], we are interested in the potential social benefits of a child’s *successful* peer play interactions. In keeping with other literatures emphasising the unique developmental contribution that peer interactions can make to children’s socio-emotional development, we hypothesise that the ability to successfully play with peers has potential to contribute to mental health over and above more general play experiences or abilities, for example play with parents or siblings [20–22]. As illustrated schematically in Fig. 1, we propose two main routes for peer play ability to influence mental health over developmental time, firstly via honing socio-cognitive competencies and secondly by developing skills in self-regulation. These skills help children form high-quality peer relational networks, that include stable connections and reciprocal friendships. Positive reciprocal relationships contribute towards lower risk for later mental health issues [1, 12]. Although we do not test all steps in these routes in the present study, we present them here as an underpinning theory, justifying the testing of associations between early peer play ability and mental health in middle childhood. We discuss each route in turn before presenting the main focus and hypotheses of the present study.

## Peer Play, Socio-cognitive Competencies, and Mental Health

The first dimension of interest is the role that peer play ability may have in supporting development of social cognition, including skills such as theory of mind and emotion recognition. Meta-analyses across clinical conditions have demonstrated a relation between social cognitive skills and psychiatric illness [23]. Social cognitive skills are important foundations for building the friendships and social support networks that have a key role in mental health [1]. While these skills that support relationship development may emerge within in the context of early parent/caregiver interactions [24, 25], we argue that peer play may be a particularly enriching context for relationship development skills to gain sophistication as children mature.

Peer play requires participating children to engage in perspective taking and to deploy their theory of mind and emotion recognition skills. To successfully engage, a child must notice who is in the mood to play, initiate or respond appropriately to a playful overture and navigate the often-unspoken terms of interaction (e.g. not hitting too hard when play fighting). Thus, peer play may provide a highly motivating opportunity to develop these sociocognitive skills to a higher level which can then be used in new contexts, for example when making new friends, resolving disputes with classmates, or sustaining existing friendships [26]. Cross-sectional research has found links between theory of mind and successful co-operation in free-play [27] and in pretend play with peers [28]. Furthermore, peer play may provide a safe and comfortable space for children to express, experience and interact with different emotions, facilitating their emotion recognition skills [29].

## Peer Play, Self-regulation, and Mental Health

A second area of development which has potential to be enhanced through peer play is that of self-regulation [30]. Regulatory skills are important for positive mental health development as they help to balance over-controlled and under-controlled attention, emotions and behaviours. Children who are more reactive face higher risks of both internalising and externalising problems. That is, children who get more easily irritated tend to exhibit more aggressive behaviours like hitting but these children can also suffer from social withdrawal and anxiety [31].

We argue that peer play could foster children's self-regulation of attention, emotion and behaviour. As discussed above, peer interactions naturally elicit effective opportunities for social communicative skills, such as turn taking and joint attention. However, at the same time, they create opportunities for disputes and negotiations among children over possible choices of props, competition for toys, different opinions over the given rules and so on [32]. Thus, self-regulatory skills enhanced during peer play may also contribute to the number and strength of social bonds in a child’s network, which in turn offer protective effects against the likelihood of mental health difficulties [22, 24].

We also draw attention to the ‘loop’ in our diagram – the arrow from high-quality peer relation networks back into play opportunities. Those with stronger social cognition, self-regulatory skills and good peer networks are likely to have more opportunities to play. Conversely, those who experience early difficulties with these aspects of development may experience fewer play opportunities and consequently further lack the experiences that help them to build positively upon on the social cognition, self-regulation, and relationships that they do have.

## The Present Study

Our research is undertaken in the broader context of building strength in the early years for positive mental health development in the long run, and we control for other bio-psycho-social factors known to be influential upon mental health risk including SES, verbal abilities, child temperament and maternal distress [2]. We also include measures of play with parents and number of siblings to control for general play experiences/opportunities. Furthermore, we include a measure of peer relationship difficulties to account for any potential restriction of such difficulties upon play opportunities [18].

We use longitudinal SEM to determine whether there is support for the general hypothesis that peer play ability in the early years may act as a protective factor against risk of internalising and externalising mental health problems in mid-childhood. Given the constraints of the secondary data available to us, we are not able in this preliminary study to test the hypothesised mediators present in our model (i.e. social cognition and self-regulation, see Fig. 1). However, a key research objective is to explore the predictive power of peer play ability upon mental health using freely available cohort data, before deciding whether more costly studies collecting data on possible mediators would be a worthwhile endeavour.

Our first research question concerns whether there is evidence for an effect of early peer play ability on later mental health outcomes at the general population level and the second research question extends this to look for evidence of protective effects of peer play in mental health high-risk groups identified by child temperamental traits at age 3 years; namely those children with low levels of persistence and those children with high levels of reactivity. We focus upon these subgroups since these temperamental traits of persistence and reactivity have been linked to both mental health and children's peer play ability [33, 34].

The specific research questions are:

### RQ1

To what extent (if any) does better peer play ability at age 3 years contribute to lower risk of internalising and externalising problems at age 7 years in a general population sample?

### RQ2

Is there evidence to support the idea of a protective effect of peer play ability age 3 years upon risk of internalising and externalising problems at age 7 years in at-risk sub-populations (a) low levels of persistence and (b) high levels of reactivity?

We hypothesise that peer play ability reduces risk of poor mental health for both the general population and the high-risk subgroups. If we find effects for early peer play ability in addition to effects for other known influences, we suggest that this will provide evidence of peer play acting as a nurturing context where children can deploy positive relational skills and build social and self-regulatory competencies. If early peer play does not explain any variance in outcomes, then this would not support the idea that there is something particularly unique about the developmental opportunities afforded by peer play, when considering influences on mental health (although of course this would not refute it). For the higher risk sub-groups, we propose that children will find it difficult to participate in and sustain peer play if they have poor persistence, or they get frustrated easily. In this case, peer play ability might not be a strong predictor of long-term mental health.

## Methods

### Participants

We used the baby cohort from the Growing Up in Australia: Longitudinal Study of Australian Children (LSAC) datasets Release 8, which tracks the cognitive and psychosocial development of children every two years in Australia. The baby cohort includes children born between March 2003 and February 2004. The sampling frame followed the Medicare Australia enrolments database and LSAC had 5107 study children in wave 1, 4606 children retained in wave 2, 4386 in wave 3 and 4242 children retained in wave 4 [35]. Even though 83% of children retained across wave 1 and 4, children who retained till wave 4 had significantly higher levels of socioeconomic status (SES) at birth, fewer children who spoken languages other than English at home and fewer children of indigenous status as illustrated in Table S1 (see supplementary materials).

In addition to attrition over time, the loss in sample size was primarily due to availability of carers’ reports on play at wave 2. Questionnaires regarding childcare quality were sent to home-based and centre-based child carers only if they provided more than 8 hours of care per week [36]. 533 eligible home-based carers and 1143 eligible centre-based carers returned questionnaires [35]. As carers’ reports were not missing at random and owing to our main research interest regarding play, we adopted full information maximum likelihood method to account for any information missing at random for these 1676 participants. This selected sample had lower proportion of children exposed to other languages at home (7.5% vs 10.8%) and fewer children of indigenous status (3.2% vs 4.5%). The selected sample also had significantly higher levels of socioeconomic status at social origin at p < 0.001 level (in Table S1 in supplementary materials). This limitation is explored in the discussion section.

### Key Measures

#### Demographic Variables

Gender, language(s) spoken at home and indigenous status were reported by parents at birth and coded into dichotomous variables. Reference categories were males, speaking languages other than English at home and not having indigenous status. The socioeconomic index was a composite measure based on parent's years of education, income and occupational prestige and it was generated by LSAC [37]. Number of siblings were reported by parents at age 3. Age in months was reported by parents.

#### Peer Play Ability at Age 3

Peer play ability was a latent factor extracted from four observed measures, i.e. how well the study child performed: *simple peer play* including give-and-take games like rolling a ball back and forth; *peer pretend play* including the use of props to play games such as dressing up and playing house; *goal-directed play* that requires collaborative efforts, to build a tower etc.; *games with rules* such as hide-n-seek and duck-duck goose. They were each rated by carers on a 3-point scale: 1 = "doesn't do it at all", 2 = "does it, but not well" and 3 = "does it well". Exploratory factor analysis extracted one factor which explained 54% of the total variance and parallel analysis of the scree plot also corroborated the extraction of one factor out of these four peer play variables (as shown in Figure S1 and Table S2 in supplementary materials).

#### Temperamental Traits at Age 3

Mothers reported children’s level of persistence and reactivity on a six-point frequency scale, which was adopted from the Australian Temperament Project [38]. There were five items on persistence, including the study child continues the same activity after a brief interruption like getting snacks; plays continuously for more than 10 min at a time with their favourite toy; stays with a routine task for 5 min or more; stops to examine objects thoroughly for 5 min or more; the child practises a new skill for 10 or more minutes. Persistence had acceptable internal consistency (Cronbach’s alpha = 0.74). There were four items on reactivity that assessed children’s propensity to react towards frustrations, including the study child responds to frustrations intensely such as yelling and screaming; has moody “off” days when he/she is irritable all day; shows much bodily movement (stomps, writhes, swings arms) when upset or crying; reacts strongly (cries, screams) when unable to complete a play activity. It had acceptable internal consistently (Cronbach’s alpha = 0.76). Scores were summed on each temperamental trait so higher scores suggest higher levels of persistence and reactivity.

In line with previous research using LSAC, we categorised children into high-risk groups for mental health problems by extracting the low-persistence subgroup from the bottom 20% of the population based on persistence scores, and the high-reactivity subgroup from the top 20% of the population based on reactivity scores [39]. We also ran Wilcoxon rank sum tests (in Table 1), which confirmed that high-risk groups identified at age 3 had significantly higher mean risks of both internalising and externalising problems at age 7.

**Table 1:** Descriptive statistics

Variables (score range)	All children in the sample N = 1676 Mean (SD)/prevalence (%)	Children not in high-reactivity group N = 1433 Mean (SD)/prevalence (%)	Children in high-reactivity group N = 243 Mean (SD)/prevalence (%)	Chi-square (df)/effect size [95% confidence interval]	Children not in low-persistence group N = 1,406 Mean (SD)/prevalence (%)	Children in low-persistence group N = 270 Mean (SD)/prevalence (%)	Chi-square (df)/effect size [95% confidence interval]
Birth variables							
Gender (male:female)	51.8%:48.2%	51.1%:48.9%	56%:44%	1.8 (1)	51.7%: 48.3%	52.2%: 47.8%	0.008 (1)
Exposure to languages not English at home (Other languages: English only)	7.5%: 92.5%	7.7%: 92.3%	5.8%: 94.2%	0.27 (1)	7.5%: 92.5%	7%: 93%	0.03 (1)
Indigenous status (Indigenous status: not indigenous status)	3.2%: 96.8%	3.4%: 96.6%	2.5%: 97.5%	0.92 (1)	3.5%: 96.5%	1.9%: 98.1%	1.45 (1)
Socioeconomic index (− 4.26–3.08)	0.23 (0.98)	0.24 (0.98)	0.16 (0.98)	0.03 [− 0.02, 0.08]	0.2 (0.98)	0.33 (0.98)	− 0.05 [− 0.1, − 0.003]*
Age 3 variables							
Simple peer play (1–3)	2.75 (0.54)	2.76 (0.52)	2.68 (0.62)	0.04 [− 0.01, 0.1]	2.76 (0.53)	2.69 (0.6)	0.04 [− 0.013, 0.087]
Pretend peer play (1–3)	2.72 (0.53)	2.78 (0.53)	2.68 (0.64)	0.06 [0.01, 0.12]*	2.77 (0.54)	2.73 (0.57)	0.03 [− 0.03, 0.08]
Goal-directed peer play (1–3)	2.76 (0.55)	2.74 (0.51)	2.61 (0.62)	0.08 [0.03, 0.13]**	2.74 (0.52)	2.65 (0.59)	0.06 [0.007, 0.11]*
Game with rules peer play (1–3)	2.51 (0.69)	2.52 (0.68)	2.44 (0.74)	0.03 [− 0.02, 0.08]	2.53 (0.68)	2.39 (0.76)	0.06 [0.01, 0.12]*
Reactivity (4–24)	12.2 (3.76)	10.86 (2.74)	17.84 (1.93)	− 0.6 [− 0.62, − 0.57]***	12.03 (3.73)	12.64 (3.86)	− 0.06 [− 0.1, − 0.008]*
Persistence (6–30)	21.3 (3.7)	21.42 (3.63)	20.88 (3.99)	− 0.04 [− 0.01, 0.08]	22.71 (2.59)	15.96 (2.13)	0.52 [0.59, 0.65]***
Number of siblings (0–8)	1.07 (0.88)	1.08 (0.87)	1.03 (0.88)	0.02 [− 0.03, 0.06]	1.08 (0.87)	1.04 (0.88)	0.02 [− 0.03, 0.07]
Family play experiences (0–3)	1.97 (0.62)	1.97 (0.63)	1.98 (0.57)	− 0.003 [− 0.004, 0.04]	1.99 (0.62)	1.88 (0.62)	0.07 [0.01, 0.11]**
Peer relationships difficulties (5–20)_	7.34 (2.55)	7.15 (2.42)	8.21 (2.97)	− 0.13 [− 0.17, − 0.07]***	7.2 (2.46)	7.93 (2.83)	− 0.09 [− 0.14, − 0.04]***
Language ability (1–110)	62.2 (25.7)	62.85 (25.3)	59.04 (27.35)	0.04 [− 0.01, 0.09]	64.02 (25.35)	54.56 (25.8)	0.13 [0.09, 0.18]***
Maternal psychological distress (6–29)	8.94 (3.13)	8.77 (2.98)	9.9 (3.72)	− 0.12 [− 0.17, − 0.07]***	8.86 (3.07)	9.36 (3.4)	− 0.06 [− 0.11, − 0.02]**
Age 7 variables							
Hyperactivity (0–10)	3.58 (2.43)	3.43 (2.36)	4.44 (2.66)	− 0.14 [− 0.19, − 0.09]***	3.41 (2.35)	4.42 (2.65)	− 0.14 [− 0.19, − 0.09]***
Conduct problems (0–9)	1.56 (1.45)	1.44 (1.39)	2.25 (1.58)	− 0.19 [− 0.23, − 0.14]***	1.46 (1.38)	2.04 (1.64)	− 0.14 [− 0.18, − 0.08]***
Emotional problems (0–9)	1.69 (1.74)	1.61 (1.7)	2.18 (1.87)	− 0.11 [− 0.17, − 0.07]***	1.66 (1.71)	1.86 (1.84)	− 0.04 [− 0.08, 0.02]
Peer problems (0–9)	1.29 (1.52)	1.24 (1.49)	1.54 (1.64)	− 0.07 [− 0.11, 0.02]**	1.23 (1.45)	1.57 (1.77)	− 0.06 [-0.11, − 0.007]*
Child’s age in months (73–99)	82.1 (3.59)	82.11 (3.61)	81.91 (3.46)	0.02 [− 0.02, 0.07]	82.1 (3.58)	81.99 (3.6)	0.009 [− 0.04, 0.06]

*p < 0.05, **p < 0.01, ***p < 0.001

#### Family Play Experiences at Age 3

Family play experiences were reported by parents. They were asked in the past week, the number of days that parents or other adults in the family engaged in arts activities like making crafts, music activities like playing music or dancing, playing toys or games indoor and playing outdoor games with the study child. We averaged the number of days across these activities.

#### Peer Relationship Difficulties at Age 3

Children’s peer relationship difficulties were reported by parents using the Pediatric Quality of Life inventory [40]. This is a 5-point scale with 1 as ‘never’ to 5 as ‘almost always’. Parents were asked in the past month, how often their child had problems playing with other children; other children not wanting to play with them; getting teased by other children; not being able to do things that other children their age could do; keeping up when playing with other children. This scale had a Cronbach’s alpha of 0.77. We summed the scores so that higher scores indicate more difficulties in peer relationships.

#### Maternal Psychological Distress at Age 3

Maternal psychological distress was assessed by summing the Kessler K6 screening scale [41]. The scale had good internal consistency (Cronbach’s alpha = 0.82). We reverse coded the scores so higher scores indicate higher levels of non-specific psychological distress.

#### Language Ability at Age 3

MacArthur Communicative Development inventories (MCDI) on vocabulary and grammar [42] were reported by mothers and were used to assess language ability at age 3. Higher MCDI scores reflect better early language and communication development.

#### Mental Health Outcomes at Age 7

Potential mental health problems are reflected by children's levels of hyperactivity, conduct, emotional and peer problems during transitioning to primary school. They were assessed by the Strength and Difficulty Questionnaire (SDQ) [43] with five items for each four sub-scales. Caregivers rated on a 3-point scale and missing items were accounted for by LSAC [44], so each sub-scale was rescaled between 0 and 10. The higher the score, the more likely the child struggled with potential mental health adversities. The hyperactivity subscale had good internal consistency (Cronbach’s alpha = 0.8). The Cronbach’s alpha for other three subscales were relatively poorer but not unacceptable, i.e. 0.58 for conduct problems, 0.65 for emotional problems and 0.59 for peer problems.

### Statistical Analyses

We conducted structural equation modelling (SEM) by using R Studio Version 1.3.1073 Lavaan package. We ran SEM analyses to examine the relations between peer play ability at age 3 and mental health outcomes at age 7 for the whole sample and high-risk sub-samples. We controlled for independent variables assessed at birth (socioeconomic status, gender, languages spoken at home and indigenous status), age 3 (peer play ability, persistence, reactivity, family play experiences, peer relationship difficulties, language ability, maternal psychological distress and number of siblings) and age 7 (age in months). Our model fit evaluations include: p-value of chi-square likelihood-ratio > 0.05, CFI > 0.95, RMSEA < 0.08 and SRMR < 0.08, but we primarily relied on the CFI, RMSEA and SRMR to decide model fits because chi-square is sensitive to large sample size [45].

## Results

### Preliminary Analyses

Table 1 presents the descriptive statistics for the entire sample. Table 1 also shows the mean differences between children who did and did not score in the top 20% for levels of reactivity at age 3, and the mean differences between children who did and did not score in the bottom 20% for levels of persistence at age 3. We also reported the effect size r and its 95% Confidence Interval (CI) by using Wilcoxon rank sum tests.

#### Comparison of the High-Reactivity and Low-Persistence Subgroups to the Remaining Sample

Compared to children in the high-reactivity subgroup, children who were not in the high-reactivity group scored significantly higher for age 3 peer pretend play (r = 0.06, 95% CI [0.01, 0.12], p = 0.012) and goal-directed play (r = 0.08, 95% CI [0.03, 0.13], p = 0.0013). Moreover, children who were not in the high-reactivity subgroup had lower levels of peer relationship difficulties (r = − 0.13, 95% CI [− 0.17, − 0.07], p < 0.001) and maternal psychological distress at age 3 (r = − 0.12, 95% CI [− 0.17, − 0.07], p < 0.001). Those not in the high-reactivity subgroup had lower risk of hyperactivity (r = − 0.14, 95% CI [− 0.19, − 0.09], p < 0.001), conduct problems (r = − 0.19, 95% CI [− 0.23, − 0.14], p < 0.001), emotional problems (r = − 0.11, 95% CI [− 0.17, − 0.07], p < 0.001) and peer problems at age 7 (r = − 0.07, 95% CI [− 0.11, − 0.02], p < 0.001).

Compared to children in the low-persistence subgroup, children who were not in the low-persistence subgroup scored significantly higher for age 3 goal-directed play (r = 0.06, 95% CI [0.007, 0.11], p = 0.017) and for games with rules (r = 0.06, 95% CI [0.01, 0.12], p = 0.001). They also had significantly more frequent family play experiences (r = 0.07, 95% CI [0.01, 0.11], p = 0.006) and higher levels of language abilities at age 3 (r = 0.13, 95% CI [0.09, 0.18], p < 0.001). In addition, children who were not in the low-persistence subgroup had lower levels of SES at birth (r = − 0.05, 95% CI [− 0.1, − 0.003], p = 0.04), lower levels of age 3 reactivity (r = − 0.06, 95% CI [− 0.01, − 0.008], p = 0.02), lower levels of peer relationships difficulties (r = − 0.09, 95% CI [− 0.14, − 0.04], p < 0.001) and lower levels of maternal psychological distress at age 3 (r = − 0.06, 95% CI [− 0.11, − 0.02], p = 0.009). Children not in the low-persistence subgroup also had lower risk of hyperactivity problems (r = − 0.14, 95% CI [− 0.19, − 0.09], p < 0.001), conduct problems (r = − 0.14, 95% CI [− 0.18, − 0.08], p < 0.001) and peer problems at age 7 (r = − 0.06, 95% CI [− 0.11, − 0.007], p = 0.02).

#### Correlations between Peer Play Indicators and SDQ Subscales

We also conducted correlational analyses between the four indicators of peer play abilities and the four internalising and externalising outcomes, see Table S3 and Table S4 in appendices. We observed positive and moderate correlations between the four observed measures of peer play ability at p < 0.001 level for all children in the whole sample, as well as for the high-reactivity subgroup and the low-persistence subgroup.

Each of the four peer play items negatively correlated with hyperactivity, conduct problems, emotional and peer problems at p < 0.001 level for all children in the group. However, most of the correlations were small, with r < 0.30. In contrast, for children in the high-reactivity subgroup, we only observed significant and negative correlations between the four indicators of peer play ability and hyperactivity. For children in the low-persistence subgroup, most of the significant correlations were between peer play abilities and hyperactivity, emotional and peer problems.

### Findings from Main Analyses: Part 1 Whole Sample Analyses

#### Does Peer Play Ability at Age 3 Years Predict Mental Health Outcomes at Age 7 Years? (RQ1)

**Fig. 2:**
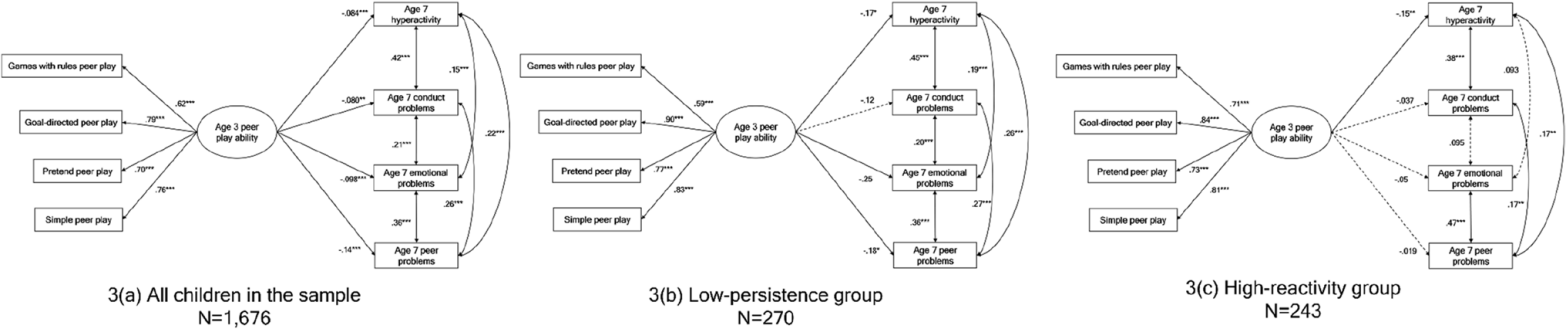
Standardised path coefficients of the relations between peer play ability at age 3 years and psychosocial outcomes at age 7 years for **a** all children in the sample; **b** children with the lowest levels of persistence at age 3; **c** children with the highest levels of reactivity at age 3. Paths coefficients for sociodemographic variables and covariates are not shown

To address this question, we present results from SEM modelling using the entire population sample (N = 1676). We modelled the effect of peer play ability on age 7 outcomes, while controlling for other factors known to influence mental health (see Fig. 2a and Table 2). The model had adequate fit with CFI = 0.951, RMSEA = 0.041, and SRMR = 0.041. The latent measure of peer play ability at age 3 years was associated with all four SDQ subscales measured at age 7 years. We present the results for each SDQ subscale in turn:

**Table 2:** Standardised SEM results for all children in the group (N = 1,676)

	Hyperactivity	Conduct problems	Emotional problems	Peer problems
*Sociodemographic backgrounds*				
Female	− 0.17 (0.026)***	− 0.054 (0.024)*	0.096 (0.025)***	− 0.015 (0.025)
Socioeconomic index at birth	− 0.14 (0.025)***	− 0.13 (0.025)***	− 0.049 (0.025)	− 0.082 (0.026)***
Home language is English	0.074 (0.029)*	0.021 (0.026)	− 0.045 (0.027)	− 0.085 (0.031)**
Indigenous status	0.048 (0.033)	0.07 (0.039)	0.016 (0.027)	0.057 (0.035)
Age in months	0.034 (0.045)	− 0.027 (0.069)	− 0.011 (0.059)	0.019 (0.067)
*Age 3 covariates*				
Peer play ability	− 0.084 (0.028)**	− 0.08 (0.028)**	− 0.098 (0.029)***	− 0.14 (0.031)***
Reactivity	0.167 (0.041)***	0.25 (0.065)***	0.12 (0.056)*	0.037 (0.066)
Persistence	− 0.2 (0.041)***	− 0.18 (0.066)**	0.002 (0.057)	− 0.045 (0.065)
Number of siblings	− 0.093 (0.025)***	− 0.027 (0.025)	− 0.082 (0.025)***	− 0.07 (0.026)**
Family play experiences	0.023 (0.025)	− 0.069 (0.025)**	− 0.035 (0.026)	− 0.054 (0.026)*
Peer relationships difficulties	0.011 (0.031)	0.042 (0.047)	0.14 (0.039)***	0.12 (0.045)**
Language ability	− 0.049 (− 0.28)	0.039 (0.473)	0.003 (0.392)	− 0.029 (0.449)
Maternal psychological distress (K6)	0.051 (0.034)	0.052 (0.057)	0.145 (0.046)**	0.13 (0.054)*

*p < 0.05, ** *p* < 0.01 *** p < 0.001. Chi-square (df) = 238.5 (62), p < 0.001. CFI = 0.951, RMSEA = 0.041, SRMR = 0.041. Standardised coefficients (standard errors) are displayed

##### Hyperactivity

Better peer play ability at age 3 (β = − 0.084, SE = 0.028, p = 0.003) was predictive of lower hyperactivity scores at age 7.

Higher persistence at age 3 (β = − 0.2, SE = 0.041, p < 0.001) and more siblings at age 3 (β = − 0.093, SE = 0.025, p < 0.001) also predicted lower hyperactivity at age 7, whereas higher reactivity at age 3 (β = 0.167, SE = 0.041, p < 0.001) predicted higher hyperactivity scores at age 7. Children’s family play experiences, peer relationships difficulties, language ability, and maternal psychological distress at age 3 were non-significant predictors.

Regarding our demographic variables, being a girl (β = − 0.17, SE = 0.026, p < 0.001) and a higher SES background at birth (β = − 0.14, SE = 0.025, p < 0.001) were predictive of lower hyperactivity scores. In contrast, children who only spoke English at home were more likely to face higher hyperactivity scores (β = 0.074, SE = 0.029, p = 0.01).

##### Conduct Problems

Better peer play ability at age 3 (β = − 0.08, SE = 0.028, p = 0.005) was predictive of lower scores for conduct problems at age 7.

Higher persistence at age 3 (β = − 0.18, SE = 0.066, p = 0.007) and more frequent family play experiences at age 3 (β = − 0.069, SE = 0.025, p = 0.006) predicted lower conduct problems scores, whereas higher reactivity at age 3 (β = 0.25, SE = 0.065, p < 0.001) predicted higher conduct problems scores at age 7. Children’s number of siblings, peer relationships difficulties, language ability and maternal psychological distress at age 3 were non-significant predictors.

Girls (β = − 0.054, SE = 0.024, p = 0.025) and children from higher SES backgrounds at birth (β = − 0.13, SE = 0.025, p < 0.001) had lower risk of conduct problems at age 7.

##### Emotional Problems

Better peer play ability at age 3 (β = − 0.098, SE = 0.029, p = 0.001) was predictive of lower emotional problems scores at age 7.

More siblings at age 3 (β = − 0.082, SE = 0.025, p < 0.001) also predicted lower emotional problems scores at age 7. In contrast, higher levels of reactivity at age 3 (β = 0.12, SE = 0.056, p = 0.038), more peer relationship difficulties (β = 0.14, SE = 0.039, p < 0.001) and higher levels of maternal psychological distress at age 3 (β = 0.145, SE = 0.046, p =  < 0.001) predicted higher scores for emotional problems at age 7. However, children’s levels of persistence, family play experiences and language ability at age 3 were non-significant predictors.

Compared to boys, girls (β = 0.096, SE = 0.025, p =  < 0.001) faced higher levels of emotional problems at age 7.

##### Peer Problems

Better play ability at age 3 (β = − 0.14, SE = 0.031, p < 0.001) was predictive of lower peer problems scores at age 7.

More siblings (β = − 0.07, SE = 0.026, p = 0.006) and more frequent family play experiences (β = − 0.054, SE = 0.026, p = 0.038) at age 3 predicted lower levels of peer problems at age 7. In contrast, more peer relationships difficulties (β = 0.12, SE = 0.045, p = 0.006) and higher levels of maternal psychological distress at age 3 (β = 0.13, SE = 0.054, p = 0.018) predicted higher scores for peer problems at age 7. Children’s levels of persistence, reactivity and language ability at age 3 were not significant predictors.

Higher SES at birth (β = − 0.082, SE = 0.026, p < 0.001) and exposure to English only at home (β = − 0.085, SE = 0.031, p = 0.006) were associated with lower levels of peer problems.

### Findings from Main Analyses: Part 2 High-Risk Subgroup Analyses

#### Does Peer Play Ability at Age 3 Years Predict Mental Health Outcomes at Age 7 Years in Children in the Low-Persistence Subgroup? (RQ2a)

To address this question, we present results from SEM modelling using the subgroup of children with the lowest levels of persistence at age 3 years (N = 270). The SEM model shown in Fig. 2b and Table 3 has adequate fit, i.e. CFI = 0.955, RMSEA = 0.047 and SRMR = 0.062.

**Table 3:** Standardised SEM results for the low-persistence subgroup (N = 270)

	Hyperactivity	Conduct problems	Emotional problems	Peer problems
*Sociodemographic backgrounds*				
Female	− 0.14 (0.06)*	0.006 (0.057)	0.079 (0.06)	− 0.049 (0.06)
Socioeconomic index at birth	− 0.11 (0.06)	− 0.18 (0.059)**	0.006 (0.06)	− 0.068 (0.061)
Home language is English	0.118 (0.08)	− 0.004 (0.059)	− 0..047 (0.064)	− 0.001 (0.061)
Indigenous status	− 0.013 (0.067)	0.043 (0.094)	− 0.087 (0.16)	0.029 (0.08)
Age in months	0.085 (0.101)	− 0.036 (0.15)	− 0.029 (0.13)	0.039 (0.14)
*Age 3 covariates*				
Peer play ability	− 0.17 (0.069)*	− 0.12 (0.067)	− 0.25 (0.071)***	− 0.18 (0.071)*
Reactivity	0.13 (0.089)	0.29 (0.13)*	0.061 (0.13)	− 0.026 (0.13)
Persistence	− 0.082 (0.063)	− 0.14 (0.084)	0 (0.075)	0.014 (0.079)
Number of siblings	− 0.16 (0.059)**	− 0.022 (0.058)	− 0.049 (0.06)	0.011 (0.061)
Family play experiences	− 0.076 (0.061)	− 0.14 (0.06)*	− 0.054 (0.061)	− 0.003 (0.061)
Peer relationships difficulties	0.11 (0.069)	0.045 (0.11)	0.102 (0.094)	0.14 (0.097)
Language ability	− 0.015 (0.6)	0.16 (0.96)	− 0.01 (0.86)	− 0.043 (0.89)
Maternal psychological distress (K6)	0.073 (0.008)	0.091 (0.13)	0.25 (0.11)*	0.26 (0.11)*

*p < 0.05, **p < 0.01, ***p < 0.001. Chi-square (df) = 98.27 (62), p = 0.002. CFI = 0.955, RMSEA = 0.047, SRMR = 0.062. Standardised coefficients (standard errors) are displayed

##### Hyperactivity

Children in the low-persistence subgroup who had better peer play ability at age 3 were more likely to have lower hyperactivity scores at age 7 (β = − 0.17, SE = 0.069, p = 0.013).

More siblings at age 3 also predicted lower hyperactivity at age 7 (β = − 0.16, SE = 0.059, p = 0.007). Children’s levels of reactivity, persistence, family play experiences, peer relationships difficulties, language ability and maternal psychological distress at age 3 were non-significant predictors of hyperactivity.

Compared to boys, girls (β = − 0.14, SE = 0.06, p = 0.024) had lower levels of hyperactivity at age 7.

##### Emotional Problems

Children in the low-persistence subgroup who had better peer play ability at age 3 were also more likely to have lower scores for emotional problems (β = − 0.25, SE = 0.071, p < 0.001) at age 7.

As expected, maternal psychological distress at age 3 (β = 0.25, SE = 0.11, p = 0.018) predicted higher levels of emotional problems at age 7.

None of the demographic factors, number of siblings, family play experiences, nor the other psychological constructs at age 3 were significant predictors of age 7 emotional problems for this subgroup.

##### Peer Problems

A similar pattern is also found for predicting levels of peer problems at age 7 faced by children with lowest level of persistence at age 3. Children with better peer play ability at age 3 had lower levels of peer problems (β = − 0.18, SE = 0.071, p = 0.012) at age 7. In contrast, maternal psychological distress at age 3 (β = 0.26, SE = 0.11, p = 0.02) predicted higher peer problems scores at age 7.

None of the demographic factors, number of siblings, family play experiences, nor the other psychological constructs at age 3 were significant predictors of age 7 peer problems for this subgroup.

##### Conduct Problems

Unlike hyperactivity, emotional and peer problems at age 7, peer play ability at age 3 was not significant in predicting age 7 conduct problems (β = − 0.12, SE = 0.067, p = 0.075) in the low-persistence subgroup. Higher levels of reactivity at age 3 significantly predicted higher scores for conduct problems at age 7 (β = 0.29, SE = 0.13, p = 0.031) for this subgroup. However, more frequent family play experiences predicted lower levels of conduct problems at age 7 (β = − 0.14, SE = 0.06, p = 0.017). Children’s level of persistence, number of siblings, peer relationships difficulties, language ability and maternal psychological distress at age 3 were not significant predictors.

Among demographic variables, only socioeconomic status at birth was associated with age 7 conduct problems (β = − 0.18, SE = 0.059, p = 0.002).

#### Does Peer Play Ability at Age 3 Years Predict Mental Health Outcomes at Age 7 Years in Children in the High-Reactivity Subgroup? (RQ2b)

To address this question, we present results from SEM modelling using the subgroup of children with highest level of reactivity at age 3 years (N = 243), the SEM model shown in Fig. 2c and Table 4 showed adequate fit, i.e. CFI = 0.959, RMSEA = 0.041 and SRMR = 0.055.

**Table 4:** Standardised SEM results for the high-reactivity subgroup (N = 243)

	Hyperactivity	Conduct problems	Emotional problems	Peer problems
*Sociodemographic backgrounds*				
Female	− 0.22 (0.07)***	− 0.054 (0.064)	− 0.055 (0.064)	− 0.15 (0.07)*
Socioeconomic index at birth	− 0.12 (0.06)	− 0.11 (0.064)	− 0.14 (0.064)*	− 0.15 (0.068)*
Home language is English	0.111 (0.099)	0.071 (0.083)	− 0.027 (0.072)	− 0.21 (0.15)
Indigenous status	− 0.023 (0.074)	0.015 (0.07)	− 0.071 (0.13)	0.02 (0.072)
Age in months	0.126 (0.111)	− 0.059 (0.17)	0.033 (0.15)	0.03 (0.16)
*Age 3 covariates*				
Peer play ability	− 0.15 (0.074)*	− 0.037 (0.071)	− 0.05 (0.073)	− 0.019 (0.07)
Reactivity	0.069 (0.065)	0.16 (0.091)	0.073 (0.077)	0.07 (0.087)
Persistence	− 0.186 (0.11)	− 0.23 (0.17)	0.07 (0.15)	− 0.074 (0.17)
Number of siblings	− 0.046 (0.062)	0.098 (0.063)	− 0.124 (0.064)	− 0.006 (0.061)
Family play experiences	0.01 (0.062)	− 0.055 (0.063)	0.002 (0.064)	− 0.073 (0.063)
Peer relationships difficulties	0.021 (0.084)	0.007 (0.136)	0.22 (0.11)*	0.2 (0.13)
Language ability	− 0.007 (0.75)	0.023 (1.25)	0.036 (1.06)	− 0.043 (1.189)
Maternal psychological distress (K6)	0.12 (0.1)	0.13 (0.17)	0.14 (0.14)	0.043 (0.162)

*p < 0.05, **p < 0.01, ***p < 0.001. Chi-square (df) = 87.34 (62), p = 0.019. CFI = 0.959, RMSEA = 0.041, SRMR = 0.055. Standardised coefficients (standard errors) are displayed

##### Hyperactivity

Children in the high-reactivity subgroup with better peer play ability at age 3 years had lower hyperactivity scores at age 7 years (β = − 0.15, SE = 0.074, p = 0.045). Children’s levels of persistence, reactivity, number of siblings, family play experiences, peer relationships difficulties, language ability and maternal psychological distress at age 3 were non-significant predictors.

Girls (β = − 0.22, SE = 0.07, p < 0.001) were more likely to have lower scores for hyperactivity at age 7.

##### Conduct Problems, Emotional Problems and Peer Problems

For the high-reactivity subgroup, there were no statistically significant differences in effect magnitudes of age 3 peer play on age 7 conduct, emotional and peer problems. Children from higher SES backgrounds at birth (β = − 0.14, SE = 0.064, p = 0.003) had lower emotional problems scores at age 7, and those children with more peer relationships difficulties at age 3 (β = 0.22, SE = 0.11, p = 0.046) were more likely to face higher levels of emotional problems at age 7. Also, both girls (β = − 0.15, SE = 0.07, p = 0.037) and children from higher SES (β = -0.15, SE = 0.068, p = 0.028) had lower age 7 peer problems scores.

### Findings from additional analyses: the influence of sibling play, family play and peer relationships difficulties on the relation between peer play and internalising/externalising problems

We also conducted mediational analyses using number of siblings, family play experiences and peer relationships difficulties to predict age 3 peer play abilities, thus examining their mediational effects on age 7 internalising and externalising problems through age 3 peer play abilities. In other words, children’s play experiences with siblings and family members, and their peer relations could potentially affect their peer play abilities at age 3, thereby affecting their mental health outcomes at age 7. There were no significant mediational effects of number of siblings, family play experiences and peer relationships difficulties via peer play abilities at age 3 for all children in the study, nor for the low-persistence and high-reactivity groups. More details can be found in Table S5 in supplementary information.

## Discussion

The current study provides evidence consistent with our hypothesis that peer play ability at age 3 years may act as a protective factor against risk of later mental health problems. Peer play ability significantly predicted lower scores on measures of hyperactivity and conduct problems (externalising), and, peer problems and emotional problems (internalising) at age 7 years, in a general population sample. Importantly, this result holds while controlling for external factors such as SES and maternal distress, and internal factors like differences in temperament. The finding also holds when potentially confounding factors are controlled; in this case number of siblings, family play experiences and peer relationship difficulties. This suggests early peer play abilities contribute to mental health risk above and beyond the child’s family circumstances and general level of socio-emotional competence. This is consistent with Toseeb and colleagues’ [9] findings but we here extend this finding to demonstrate that effects of peer play ability on later risk of externalising/internalising problems can be seen for children as young as 3 years old, and in both the general population and for children with temperamental difficulties characterised by low-persistence.

We also conducted mediational analyses and found that number of siblings, family play experiences and peer relationships difficulties did not significantly predict age 3 peer play abilities. Therefore, the effects of age 3 peer play abilities over age 7 internalising and externalising problems were independent of children’s play experiences with parents and siblings, and of any relationship problems with peers. These findings corroborate our argument that peer play competence should be valued as holding a developmentally unique and beneficial influence on children’s long-term mental health outcomes. As Smith argued [14], social play allows animals and humans to practise complex skills and improve behavioural flexibilities to adjust to new contexts. Similarly, our conceptual framework in Fig. 1 suggests that successful peer play experiences could facilitate the development of socio-cognitive and self-regulatory skills, contributing towards emotional intimacy and building higher quality links in peer networks, which further reduce risk of potential mental health problems [12, 22, 26]. In addition, peer play ability at age 3 was found to act as a protective factor for hyperactivity at age 7 for both those with low levels of persistence and those with high levels of reactivity. This is consistent with findings that peer activities can help to improve the social functioning of children with ADHD possibly via co-regulation of behaviours [46].

We also observed an effect of peer play on emotional problems for children in the lowest persistence subgroup. This may suggest that peer play may facilitate the development of problem-solving skills in children who otherwise tend to ‘give up’ on challenging situations, building essential coping abilities that predict lower risks of internalising problems for school-aged children [47]. We did not find significant predictive power of peer play on internalising problems for the high-reactivity subgroup. This is possibly because highly reactive children often struggle with social withdrawal and anxiety, thus reducing their play opportunities in the first place (as indicated by the significant influence of peer relationship difficulties in predicting emotional problems in Table 4) [18, 31].

### Peer Play at Age 3 as a Potential Screening Tool

Among all predictors of age 7 externalising and internalising problems, peer play ability at age 3 years was the only significant factor that predicted all four outcomes for the whole population, which indicates that it could serve as a screening tool for identifying children potentially at-risk for developing long-term psychopathology. Maternal psychological distress at age 3 was significant in predicting internalising problems for the whole population but not externalising problems at age 7 years. This finding is in line with meta-analyses that show consistent links between maternal depression and children's negative emotionality [2]. Another prominent factor is socioeconomic status (SES) at birth. Our results showed that children from higher SES families at birth tend to face lower risks of hyperactivity, conduct problems and peer problems but it was not significant in predicting emotional problems at age 7. This is in line with research on the strong link between poverty and poor mental health problems during childhood [48]. Number of siblings was a proxy for sibling play opportunities, which only significantly predicted risks of hyperactivity and internalising problems, which again demonstrates the importance of peer-level influence that is beyond family-level influence [49]. Taken together we suggest that future research could explore whether questions about peer play ability could be included in case-history taking or screening designed to identify those children most at-risk of poor mental health outcomes.

### Peer Play at Age 3 as a Potential Intervention Target

Drawing from Alper and colleagues’ argument [50] that parent–child play can provide a natural and enjoyable route for interventions because it may be more readily changed than more entrenched risk factors, we argue that peer play ability is likely more malleable compared to risk factors such as maternal psychological distress and poverty. It is arguably more challenging to mitigate the negative impacts of these major risk factors than to intervene to foster children’s peer play skills. This is also true for children with high levels of reactivity and children with low level of persistence at age 3. The findings about at-risk groups suggest the possibility of improving children’s future prospects of mental health by strengthening their peer play ability at age 3. Peer interventions have gained in popularity in recent decades as they improve children's adaptive functioning by teaching key social skills such as communication and cooperation via activities such as games and sports [51]. For example, a recent systematic review demonstrated moderate effects of interventions that incorporated peer play skills such as peer role play and games on improving the concurrent and long-term social functioning of children with ADHD [52]. For instance, Wilkes-Gillan and colleagues [53] found that a 10-week peer play intervention targeting cooperative play skills in children with ADHD and their playmates led to significant gains in social skills, including perspective taking and understanding verbal and non-verbal cues, which were maintained after 18 months [54]. These skills are important for children with ADHD to develop more positive peer relations [55].

Our findings also highlight that peer play abilities in engaging with peer pretend play, goal-directed and rule-governed peer play contexts should be explored as low-cost interventions that could be delivered outside of mental health services and easily adapted to home and preschool contexts. We argue that it is particularly useful to consider the potential of promoting peer play skills especially against the backdrop of enduring inequalities in access to psychiatric services around the world, which urgently demands more universal, preventative routes to mental health support. Peer play based interventions have great potential since they are easily accessible, low-cost and adaptable across various socioeconomic contexts [56].

### Limitations

There are some limitations to consider when interpreting our findings. Although our sample is large, children retained from wave 1 to 4 in LSAC tended to come from higher SES and less diverse sociodemographic backgrounds. The sample was again reduced due to peer play measures being reported by carers. This further biased the sample towards higher-income families because children with carers available to report play measures had higher SES at birth. Future studies could deliberately over-sample lower SES groups to address this imbalance and check if findings hold across a wider SES spectrum.

The three-point peer play scale was reported by carers, who observe the child on a regular basis and for at least 8 hours per week. Even though it did not offer as detailed information on peer play ability as measures like Parten’s categorisation of peer play, we argue that this scale could reflect more consistent and ecologically valid levels of children’s peer play abilities since scales like Parten’s are often based upon one single peer play episode. In addition, there were between 4 and 12% of children in our sample who did not engage with either simple peer play, peer pretend play, goal-directed peer play or rule-governed peer play at all. Rubin and colleagues [57] adopted similar measures on children aged between 45 and 59 months but instead of ratings, they calculated the number of seconds children spent on these four types of peer play within a 30-min video observation. They found that children spent 6.57% of the time being unoccupied and 12.43% of the time being onlookers. These percentages are quite similar to ours, potentially suggesting that the three-point peer play scale was portraying what should be expected from a sample of children at around this age. However, we acknowledge that our study is one of the first to use this scale. Since there is no publicly accessible information on the rationale behind it, we call for future research to validate the measure.

In addition, as our control for general play experiences, we only had number of siblings and frequency of family play activities, which offer information on the quantity rather than quality of play. Thus, we call for future research to explore the quality of parent–child play and sibling play in relation to mental health outcomes.

There are also areas that we did not explore in the current study, including hours spent in childcare, and whether children engaged in peer play with the same partner consistently or with a variety of other children. In addition, we explored the influence of temperamental traits on peer play abilities, but we did not explore another possible direction, i.e. that peer play abilities contribute towards temperamental traits, thereby affecting long-term mental health development. Having established the predictive effects of peer play abilities on mental health, while accounting for temperamental factors, we suggest that future studies could use experimental designs to tease apart key dependencies.

As discussed above, we proposed the potential links between peer play abilities and sociocognitive, self-regulatory and problem-solving skills but were unable to testify them empirically as we are constrained by the secondary data available. Given our results we conclude that our hypothesised model of a protective effect for early peer play is worth exploring in more depth. The current study does not intend to draw causal relations due to our non-experimental design. However, by establishing longitudinal relations between peer play and mental health outcomes, our study provides supporting evidence for theories suggesting positive effects of peer play ability on children’s long-term mental health development. This evidence is more robust than a simple cross-sectional association and provides an important indicative piece of evidence suggesting more costly randomised studies in this area are worth pursuing further.

## Summary

The present study corroborates the idea of a potential protective role of peer play at age 3 years for both externalising and internalising problems at age 7 years. Peer play at age 3 also holds predictive power over certain mental health outcomes for children exhibiting high levels of reactivity and low levels of persistence at age 3. To our knowledge, the present paper is the first longitudinal cohort analysis that provides empirical evidence for the protective effects of peer play in children as young as 3 years old. Our findings represent an important new direction for development of public health approaches to mental health in infancy/early childhood. We encourage future work on these hypotheses to further explore the underlying relations between peer play and mental health outcomes, particularly for children at-risk for mental health disorders.

### Supplementary Information

Below is the link to the electronic supplementary material.

Supplementary file1 (DOCX 132 kb)
