# Supplementary material for: Evidence for Protective Effects of Peer Play in the Early Years: Better Peer Play Ability at Age 3 Years Predicts Lower Risks of Externalising and Internalising Problems at Age 7 Years in a Longitudinal Cohort Analysis
Source: Child Psychiatry Hum Dev. 2022 Jun 14;54(6):1807–22. doi: 10.1007/s10578-022-01368-x (PMC10581935; doi:10.1007/s10578-022-01368-x)
Supplement: Supplementary file 1 — Supplementary file1 (DOCX 132 kb) [file 10578_2022_1368_MOESM1_ESM.docx]

**Supplementary materials for Zhao& Gibson Peer Play**

Contents

[**Supplementary tables** 2](#_Toc99980536)

[**Table S1** 2](#_Toc99980537)

[*Descriptive statistics of demographic factors for selected sample and full population at wave 1* 2](#_Toc99980538)

[**Table S2** 3](#_Toc99980539)

[*Exploratory factor analysis results for all children in the sample* 3](#_Toc99980540)

[**Table S3** 4](#_Toc99980541)

[*Correlations between key measures for full population* 4](#_Toc99980542)

[**Table S4** 5](#_Toc99980543)

[*Correlations between key measures for low-persistence and high-reactivity groups* 5](#_Toc99980544)

[**Table S5** 7](#_Toc99980545)

[*Mediational analyses on sibling play/family play/peer relationships difficulties, peer play abilities and internalising/externalising problems* 7](#_Toc99980546)

[**Supplementary figures** 8](#_Toc99980547)

# **Supplementary tables**

## **Table S1**

## *Descriptive statistics of demographic factors for selected sample and full population at wave 1*

|  | **Retained sample between wave 1 to 4** | | **Selected sample** | | **Wave 1 full population** | |
| --- | --- | --- | --- | --- | --- | --- |
| **Variables** | **N** | **Mean (SD)/ prevalence (%)** | **N** | **Mean (SD)/ prevalence (%)** | **N** | **Mean (SD)/ prevalence (%)** |
| Gender (male: female) |  |  |  |  |  |  |
| Male | 2187 | 51.6%: | 868 | 51.8%: | 2608 | 51.1% |
| Female | 2055 | 48.4% | 808 | 48.2% | 2499 | 48.9% |
| Exposure to languages not English at home |  |  |  |  |  |  |
| Other languages | 399 | 9.4% | 125 | 7.5% | 552 | 10.8% |
| English Only | 3843 | 90.6% | 1551 | 92.5% | 4555 | 89.2% |
| Indigenous status |  |  |  |  |  |  |
| Indigenous status | 145 | 3.4% | 54 | 3.2% | 230 | 4.5% |
| Not indigenous status | 4097 | 96.6% | 1622 | 96.8% | 4877 | 95.5% |
| Socioeconomics index at birth | 1675 | 0.09 (0.98) | 1675 | 0.23 (0.98)*** | 4088 | 0.00 (1) *** |
| **Total number of observations** | 4242 |  | 1676 |  | 5107 |  |

*Note.* *** *p* < 0.001 for Wilcoxon rank sum tests between retained sample (wave1 to 4) and selected sample; between retained sample (wave1 to 4) and wave 1 full population

## **Table S2**

## *Exploratory factor analysis results for all children in the sample*

|  | **Factor loadings** | **Commonalities** |
| --- | --- | --- |
| Simple peer play | 0.76 | 0.58 |
| Pretend peer play | 0.72 | 0.52 |
| Goal-directed peer play | 0.79 | 0.63 |
| Game with rules peer play | 0.65 | 0.42 |

*Note. SS loading was 2.14; proportion of variance explained was 54%; root mean square of the residuals was 0.03.*

## **Table S3**

## *Correlations between key measures for full population*

|  | **1** | **2** | **3** | **4** | **5** | **6** | **7** | **8** | **9** | **10** | **11** | **12** | **13** | **14** | **15** | **16** |
| --- | --- | --- | --- | --- | --- | --- | --- | --- | --- | --- | --- | --- | --- | --- | --- | --- |
| 1. **SES at birth** | 1 |  |  |  |  |  |  |  |  |  |  |  |  |  |  |  |
| 1. **Simple peer play** | .03 | 1 |  |  |  |  |  |  |  |  |  |  |  |  |  |  |
| 1. **Peer pretend play** | .04 | .5*** | 1 |  |  |  |  |  |  |  |  |  |  |  |  |  |
| 1. **Goal-directed peer play** | .06* | .61*** | .56*** | 1 |  |  |  |  |  |  |  |  |  |  |  |  |
| 1. **Rule-governed peer play** | .01 | .41*** | .5*** | .46*** | 1 |  |  |  |  |  |  |  |  |  |  |  |
| 1. **Persistence** | .03 | .1*** | .05 | .11*** | .11*** | 1 |  |  |  |  |  |  |  |  |  |  |
| 1. **Reactivity** | -.1*** | -.09** | -.13*** | -.13*** | -.1*** | -.1*** | 1 |  |  |  |  |  |  |  |  |  |
| 1. **Number of siblings** | -.02 | -.02 | .03 | <.001 | -.01 | -.04 | <.001 | 1 |  |  |  |  |  |  |  |  |
| 1. **Family play experiences** | .1*** | <.001 | -.05 | .03 | -.01 | .15*** | -.04 | -.16*** | 1 |  |  |  |  |  |  |  |
| 1. **Peer relationships difficulties** | .04 | -.07* | -.1*** | -.06 | -.1** | -.18*** | .21*** | .06* | .02 | 1 |  |  |  |  |  |  |
| 1. **Maternal distress** | -.11*** | -.04 | -.05* | -.07** | -.07** | -.14*** | .22*** | .03 | -.04 | .2*** | 1 |  |  |  |  |  |
| 1. **Language abilities** | .11*** | .11 | .11*** | .13*** | .16*** | .2*** | -.13*** | -.04 | .15*** | -.1*** | -.05 | 1 |  |  |  |  |
| 1. **Hyperactivity** | -.18*** | -.1*** | -.12*** | -.17*** | -.08** | -.24*** | .23*** | -.07** | -.03 | .1*** | .13*** | -.14*** | 1 |  |  |  |
| 1. **Conduct problems** | -.18*** | -.11*** | -.1*** | -.14*** | -.09*** | -.23*** | .31*** | <.001 | -.11*** | .13*** | .16*** | -.09** | .5*** | 1 |  |  |
| 1. **Emotional problems** | -.08** | -.09*** | -.11*** | -.1*** | -.07* | -.07* | .19*** | -.06* | -.03 | .19*** | .21*** | -.05 | .19*** | .27*** | 1 |  |
| 1. **Peer problems** | -.12*** | -.12*** | -.16*** | -.14*** | -.12*** | -.11*** | .12*** | -.05 | -.06* | .18*** | .19*** | -.09** | .28*** | .33*** | .41*** | 1 |

*Note. * p* < 0.05, ** *p* < 0.01, *** *p* < 0.001

## **Table S4**

## *Correlations between key measures for low-persistence and high-reactivity groups*

|  | **1** | **2** | **3** | **4** | **5** | **6** | **7** | **8** | **9** | **10** | **11** | **12** | **13** | **14** | **15** | **16** |
| --- | --- | --- | --- | --- | --- | --- | --- | --- | --- | --- | --- | --- | --- | --- | --- | --- |
| 1. **SES at birth** | 1 | .14* | .1 | .04 | 04 | -.03 | -.06 | .09 | .02 | .01 | -.13 | .01 | -.17* | -.14* | -.17* | -.18** |
| 1. **Simple peer play** | .12 | 1 | .55*** | .7*** | .61*** | .26*** | -.04 | -.02 | -.06 | -.11 | -.08 | .1 | -.19** | -.13 | -.06 | -.07 |
| 1. **Peer pretend play** | .07 | .63*** | 1 | .63*** | .6*** | .09 | -.08 | .07 | -.1 | -.08 | -.02 | .08 | -.18* | -.02 | -.06 | -.05 |
| 1. **Goal-directed peer play** | .07 | .75*** | .7*** | 1 | .56*** | .22*** | .01 | .03 | -.11 | -.06 | -.06 | .14* | -.21** | -.14* | -.08 | -.09 |
| 1. **Rule-governed peer play** | .02 | .51*** | .5*** | .53*** | 1 | .29*** | -.12 | <.001 | -.06 | -.12 | -.06 | .1 | -.18* | -.11 | -.07 | -.11 |
| 1. **Persistence** | .11 | .15* | .12 | .2** | .14* | 1 | .04 | -.17** | .03 | -.19** | -.23 | .11 | -.26*** | -.3*** | .01 | -.15* |
| 1. **Reactivity** | -.07 | -.22*** | -.2** | -.24*** | -.22** | -.13* | 1 | -.09 | .03 | -.01 | .16* | -.14* | .11 | .16* | .12 | .09 |
| 1. **Number of siblings** | -.01 | -.04 | .02 | -.01 | -.02 | -.15* | .06 | 1 | -.03 | .1 | .1 | .01 | -.02 | .12 | -.12 | .01 |
| 1. **Family play experiences** | .11 | .04 | -.06 | <.001 | -.12 | <.001 | .04 | -.2*** | 1 | .12 | .08 | .11 | .03 | -.05 | .04 | -.05 |
| 1. **Peer relationships difficulties** | -.01 | -.12 | -.14* | -.14* | -.2** | -.17** | .17* | .12 | .11 | 1 | .22*** | -.16* | .08 | .07 | .23*** | .28*** |
| 1. **Maternal distress** | -.18** | -.16* | -.16* | -.15* | -.12 | -.13* | .28*** | .04 | .07 | .22*** | 1 | -.15* | .19** | .23*** | .18** | .16* |
| 1. **Language abilities** | .13* | .15* | .13* | .14* | .23*** | .14* | -.11 | -.13* | .15* | -.07 | -.03 | 1 | -.08 | -.09 | <.001 | -.11 |
| 1. **Hyperactivity** | -.17** | -.21*** | -.19*** | -.29*** | -.07 | -.13* | .25*** | -.07 | -.05 | .18** | .19** | -.07 | 1 | .45*** | .16* | .25*** |
| 1. **Conduct problems** | -.23*** | -.21*** | -.1 | -.25*** | -.14* | .21*** | .35*** | .04 | -.1 | .13* | .24*** | .02 | .51*** | 1 | .12 | .23*** |
| 1. **Emotional problems** | -.05 | -.29*** | -.22*** | -.28*** | -.11 | -.1 | .18** | -.02 | -.01 | .18** | .31*** | -.06 | .28*** | .29*** | 1 | .51*** |
| 1. **Peer problems** | -.14* | -.21** | -.15* | -.27*** | -.07 | -.09 | .13* | .06 | <.001 | .23*** | .32*** | -.08 | .25*** | .34*** | .45*** | 1 |

*Note. * p* < 0.05, ** *p* < 0.01, *** *p* < 0.001. Low-persistence group correlations are below the diagonal and in grey shade. High-reactivity group correlations are above the diagonal and without shade.

## **Table S5**

### *Mediational analyses on sibling play/family play/peer relationships difficulties, peer play abilities and internalising/externalising problems*

|  | **Hyperactivity** | **Conduct problems** | **Emotional problems** | **Peer problems** |
| --- | --- | --- | --- | --- |
| **All children in the group N=1,676** | | | | |
| **Number of siblings to peer play ability to outcome** | 0 [-0.004, 0.005] | 0 [-0.004, 0.005] | 0 [-0.005, 0.005] | 0 [-0.007, 0.008] |
| **Family play experiences to peer play ability to outcome** | 0.003 [-0.002, 0.007] | 0.002 [-0.002, 0.007] | 0.003 [-0.002, 0.008] | 0.004 [-0.003, 0.012] |
| **Peer relationships difficulties to peer play ability to outcome** | 0.012 [0, 0.024] | 0.01 [0, 0.02] | 0.007 [-0.001, 0.015] | 0.007 [-0.001, 0.015] |
| **Low-persistence group N=270** | | | | |
| **Number of siblings to peer play ability to outcome** | -0.001 [-0.023, 0.02] | -0.001 [-0.016, 0.015] | -0.002 [-0.034, 0.03] | -0.001 [-0.024, 0.022] |
| **Family play experiences to peer play ability to outcome** | -0.001 [-0.022, 0.021] | 0 [-0.016, 0.015] | -0.001 [-0.032, 0.031] | -0.001 [-0.023, 0.022] |
| **Peer relationships difficulties to peer play ability to outcome** | 0.031 [-0.002, 0.064] | 0.023 [-0.007, 0.052] | 0.041 [0, 0.082] | 0.033 [-0.001, 0.068] |
| **High-reactivity group N=243** | | | | |
| **Number of siblings to peer play ability to outcome** | -0.004 [-0.025, 0.017] | -0.001 [-0.008, 0.006] | -0.001 [-0.008, 0.006] | 0 [-0.005, 0.004] |
| **Family play experiences to peer play ability to outcome** | 0.014 [-0.011, 0.039] | 0.004 [-0.011, 0.019] | 0.004 [-0.011, 0.019] | 0.001 [-0.012, 0.014] |
| **Peer relationships difficulties to peer play ability to outcome** | 0.02 [-0.009, 0.049] | 0.006 [-0.014, 0.025] | 0.006 [-0.014, 0.025] | 0.002 [-0.016, 0.02] |

*Note. Standardised coefficients were reported. Other coefficients were reported in Table 2-4. * p* < 0.05, ** *p* < 0.01, *** *p* < 0.001.

# **Supplementary figures**


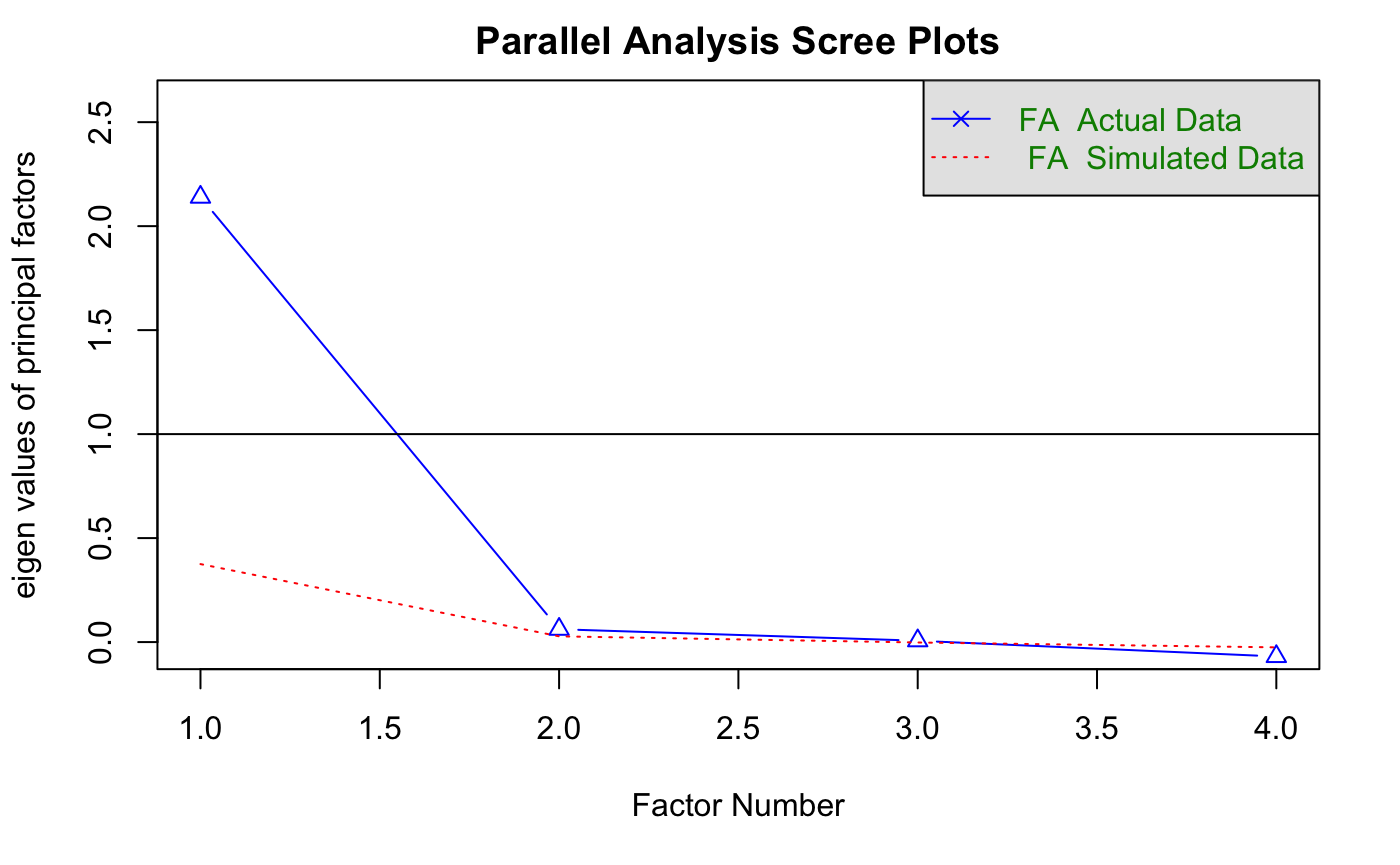


*FigureS1.* Parallel analysis scree plots for factor analysis for all children in the sample
